# Motion Streaks Do Not Influence the Perceived Position of Stationary Flashed Objects

**DOI:** 10.1100/2012/909547

**Published:** 2012-05-03

**Authors:** Andrea Pavan, Rosilari Bellacosa Marotti

**Affiliations:** ^1^Cognitive Neuroscience Sector, International School for Advanced Studies (SISSA)—Via Bonomea 265, 34136, Trieste, Italy; ^2^Department of General Psychology, University of Padua, Via Venezia 8, 35131 Padua, Italy

## Abstract

In the present study, we investigated whether motion streaks, produced by fast moving dots Geisler 1999, distort the positional map of stationary flashed objects producing the well-known motion-induced position shift illusion (MIPS). The illusion relies on motion-processing mechanisms that induce local distortions in the positional map of the stimulus which is derived by shape-processing mechanisms. To measure the MIPS, two horizontally offset Gaussian blobs, placed above and below a central fixation point, were flashed over two fields of dots moving in opposite directions. Subjects judged the position of the top Gaussian blob relative to the bottom one. The results showed that neither fast (motion streaks) nor slow moving dots influenced the perceived spatial position of the stationary flashed objects, suggesting that background motion does not interact with the shape-processing mechanisms involved in MIPS.

## 1. Introduction

Form and motion are not processed separately but they continuously interact and influence each other. Form processing can assist and facilitate the extraction of motion information: an example is given by the motion streaks generated by fast moving features [1–3]. When a feature moves fast enough (about one feature width per 100 ms [1]) it becomes smeared in space [4] owing to temporal integration and creating a spatial signal oriented towards the direction of motion (i.e., motion streak/speed line [1, 2, 5, 6]). Motion streaks are thought to be extracted by the static orientation system and combined with the output of perpendicularly oriented direction-selective detectors [1]. Such combined detector would encode both the orientation and the direction of a motion streak.

Other studies have pointed out how the information about orientation (i.e., form) is able to influence the perceived direction of moving stimuli [3, 7]. Burr and Ross [3], for example, have shown that motion streaks are exploited by our visual system to improve direction discrimination. In particular, they showed that when using an oriented noise mask with the orientation parallel to the direction of motion, direction discrimination was largely affected, whereas it was little affected when noise mask was orthogonal to motion direction.

Moreover, these mask effects did not impair contrast sensitivity and speed discrimination. Edwards and Crane [6] further showed that motion streaks assist motion detection. Lower detection thresholds were found when using a long streak (i.e., the same dot carried the motion signal across successive motion frames), with high speeds and at high contrast. Though motion streaks improve the extraction of motion information, they can also influence the spatial orientation of stationary objects. Apthorp and Alais [8], adapting to weak and strong streaks (obtained by varying the streak length), found that only strong motion streaks were able to produce a tilt aftereffect (TAE) when testing with an oriented grating. TAE was similar in strength when adapting to static oriented gratings. These results imply that motion streaks can adapt orientation-selective neurons and further suggest that the visual system has detectors that combine form and motion.

There is also evidence that motion streaks not only influence the perceived direction, speed, or orientation of a stimulus, but also affect the shape of illusory contours. Li et al. [9] have shown that when using a convex Kanizsa triangle (generated with circular missing wedge segments subtending an angle slightly greater than 60 deg) superimposed to a field of fast (6 deg/s) globally contracting moving dots (diameter: 0.09 deg = ~6 arcmin), the edges of the convex Kanizsa triangle were perceived as regular. That is, the globally contracting dots induced a distortion of the illusory edges of the Kanizsa triangle along the motion direction. These results suggest that motion streaks can affect the perceived shape of illusory objects, confirming the interaction between motion and form.

Psychophysical evidence that motion smear from fast moving elements can influence the shape of a stimulus has been provided by Khuu et al. [10]. The authors used two vertical bars moving in apparent motion (stroboscopic) and flashed two Gaussian blobs (50 ms) in the middle of the illusory motion path. They found that, at short interstimulus intervals (ISIs) between the two bars forming the illusory motion sequence and at high eccentricity, the test Gaussian blob appeared wider than the reference Gaussian blob. The authors argued that motion smear could arise from apparent motion and it is able to influence the perceived shape of a test stimulus. Previous studies have shown that apparent motion activates extrastriate areas (e.g., MT) as well as the striate cortex (V1) [11–13]. Moreover, detectors sensitive to motion parallel to the preferred orientation are present in both V1 and MT areas [2, 14, 15]. Thus, such “parallel-motion” detectors, coding information from between the two sites of stimulation in an apparent motion sequence, though they do not receive direct physical input, could be activated to produce motion smear. Motion smear could consequently affect the spatial representation of a stimulus presented between the two stimulation sites distorting its shape. Moreover, using a stimulus configuration similar to that of Khuu et al. [10], the position of the stimulus was shifted in the direction of the apparent motion (Motion Induced Position Shift-MIPS [16, 17]). These studies suggest that the smear from apparent motion can affect both the shape and the positional map of a stimulus. As Whitney et al. [18], Arnold and Johnston [19], and Tsui et al. [20] have shown, a shape elongation may result in a centroid shift in the motion direction and thus resulting in a shift of the perceived position of the stimulus in the direction of motion (MIPS). In particular, Tsui et al. [20] showed that during motion, the perceived contrast of a Gabor patch increases at its leading edge (where the motion ends) and decreases at its trailing edge (where the motion starts), causing an unbalance in the perceived contrast between the two edges of the Gabor, thus inducing a distortion of the global shape of the Gabor patch. The consequence is that the centroid of the stimulus is physically shifted in the direction of motion leading to a change in the perceived position of the whole stimulus (MIPS; see [21] for a formal model).

In the present study, we investigated whether the perceived spatial position of stationary flashed objects can be biased by other objects moving on the background. In particular, two stationary Gaussian blobs, one above and the other at the bottom of a central fixation point, were flashed over two fields of dots moving coherently in opposite directions. Subjects had to judge the relative position of the top Gaussian blob with respect to the bottom one. We tested two speeds: a fast speed that produced motion streaks and a slow speed that did not produce motion streaks. We assessed whether moving dots producing motion streaks (i.e., motion smear) can induce a bias in the perceived spatial position of stationary flashed objects.

## 2. Method

### 2.1. Subjects

Two authors and nine naïve subjects served as observers in both the experiments. All subjects had normal or corrected-to-normal visual acuity. Subjects sat in a dark room and were placed at 57 cm from the screen. Viewing was binocular. They were instructed to fixate on the center of the screen and were given initial training to familiarize with the stimuli and the task. Subjects participated voluntarily with compensation and gave their informed consent prior to their inclusion in the experiment.

### 2.2. Apparatus

Stimuli were displayed on a 19-inch CTX CRT Trinitron monitor with a refresh rate of 60 Hz and generated with Matlab Psychtoolbox [22, 23]. The screen resolution was 1280 × 1024 pixels. Each pixel subtended ~1.9 arcmin. The mean luminance was 52.8 cd/m^2^. Luminance was measured using a Minolta LS-100 photometer. A gamma-corrected lookup table (LUT) was used so that luminance was a linear function of the digital representation of the image.

### 2.3. Stimuli

**Figure 1:**
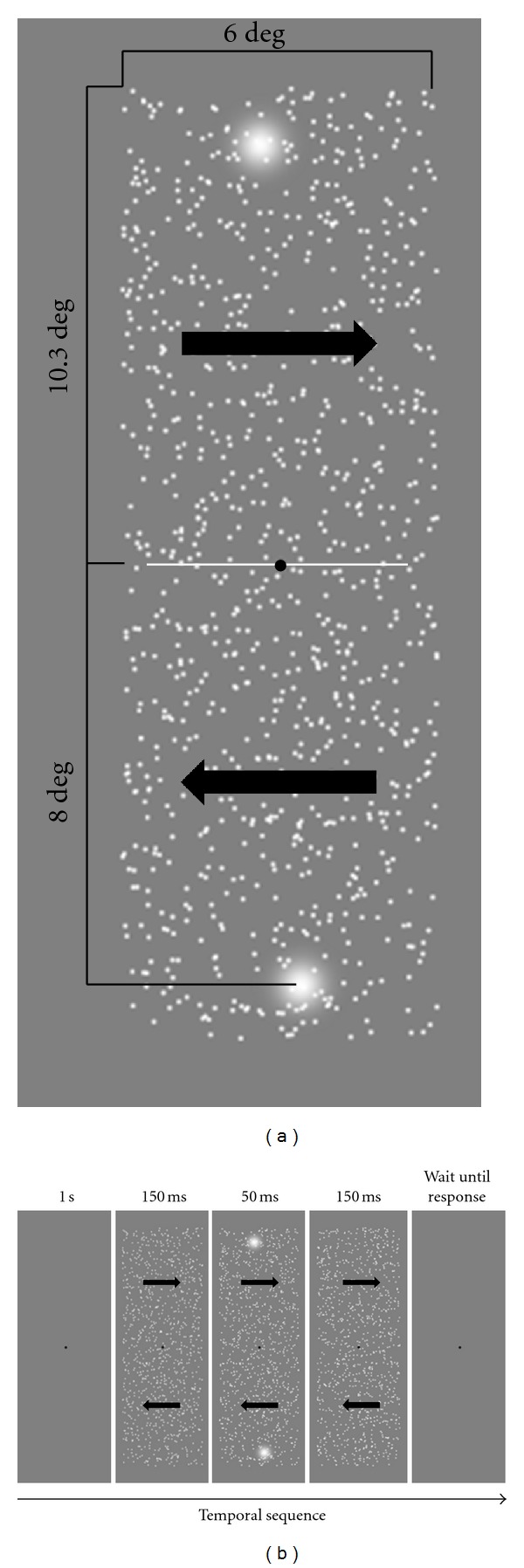
(a) Stimulus configuration used in the experiments. The figure shows the condition with coherently moving dots. Each sub-windows was 6.0 × 10.3 deg. In the example dots present in the upper subwindow move rightward, whereas dots in the bottom sub-window move rightward (thick black arrows); however we used the opposite condition as well. Gaussian blobs were flashed 8.0 deg above and below the central fixation point. In the example, the Gaussian blobs have a horizontal offset of 26.4 arcmin with respect to the fixation point. The white line that crosses the central fixation point delimits the two sub-windows, but it was not present during the experiment. We used also a condition with no moving dots and a condition in which dots moved randomly. See text for more details. (b) A schematic representation of the stimulus temporal sequence. Observers were required to fixate the fixation point at the center of the screen and after 1 s coherently moving dots were presented. In the example, dots in the upper sub-windows moved always rightward, whereas dots in the bottom sub-window moved always leftward (thick black arrows). After 150 ms two Gaussian blobs were flashed for 50 ms. It should be noted that when blobs were flashed the dots still moved. Then, moving dots continue to move for 150 ms. Each trial had a duration of 350 ms, with Gaussian blobs flashed in the middle of the temporal window. After the stimulus presentation the program waited until subject's response. The same stimulus temporal sequence was used for the conditions with no moving dots and randomly moving dots.

Stimuli consisted of a dense spatial array of 966 white dots (102.3 cd/m^2^; Weber contrast: 0.94) displayed within a rectangular window (6.0 × 23.5 deg) at the center of the screen (density: 6.83 dot/deg^2^). The background was set at the mean luminance (52.8 cd/m^2^) (Figure 1(a)). Each moving dot had a width of 0.1 deg (i.e., 6 arcmin). The dots' luminance and contrast were kept constant even when dots overlapped during motion. Dots had a speed either of 1.77 or 17.7 deg/s. According to Geisler [1], streaks start to emerge at the speed of ~2.5 deg/s for a 6 arcmin stimulus, so fast (17.7 deg/s) coherently moving dots produced motion streaks (i.e., motion smear), since they moved with a speed above one dot width per 100 ms (i.e., in the case of 17.7 deg/sec a single dot covered 1.47 deg over 100 ms), while slow coherently moving dots barely overcome one dot width per 100 ms (i.e., 0.147 deg over 100 ms) [1, 2, 6].

At the beginning of each trial an “age” value (ranging from 16.67 to 350 ms) was assigned to each dot; that is, a single dot could appear from the first frame (age: 16.67 ms) or at sometime within a temporal window ranging from the first frame to 350 ms, thus in each trial (duration: 350 ms) dots appeared asynchronously on the display. Moreover, dots had a limited lifetime: when the age of a single dot reached 350 ms, the dot vanished and was replaced by a new dot at a randomly position within the window with an age of 16.67 ms. In addition, moving dots that traveled outside the window were wrapped around to the opposite edge. Depending on the experimental condition dots could move coherently (100% coherence), either leftward or rightward, or in random directions (0% coherence); in both the displays, speed could be set to one of the speeds mentioned above (i.e., either 1.77 or 17.7 deg/s). For both the speeds in the latter display, we did not expect motion streaks (i.e., motion smear) to be generated since dots had no motion coherence. In different conditions, two test Gaussian blobs were flashed and superimposed to moving dots (see Procedure). The contrast of the test Gaussian blobs was 0.94 (Weber contrast) and the spatial constant (*σ*) was 0.42 deg [10] (Figure 1(a)).

### 2.4. Procedure

Observers fixated a point at the center of the screen and judged the relative position of two flashed Gaussian blobs placed at 8.0 deg above and 8.0 deg below the fixation point. We measured the positional shift induced by fast (17.7 deg/sec) and slow (1.77 deg/sec) moving dots. The experiment consisted of three main conditions. (i) The experimental condition: the window in which moving dots were displayed was split in two subwindows (either 6.0 × 10.3 deg), one placed above the fixation point and the other below it, in a manner that the border between the two subwindows coincided with the fixation point. The dots present in the two subwindows always moved coherently (100%) in opposite directions; that is, if the dots in the upper subwindow moved leftward, dots in the bottom subwindow moved rightward (Figure 1(a)). After an initial fixation point (1 sec), moving dots appeared for 350 ms. After 150 ms from the beginning of the motion, two test Gaussian blobs superimposed to the moving dots were flashed for 50 ms (Figure 1(b)). The two test Gaussian blobs were presented horizontally offset in opposite directions by one of five values (−26.4, −8.8, 0.0, 8.8, and 26.4 arcmin; positive values indicate rightward offset, negative values indicate leftward offset). Subjects indicated with a button press whether the top Gaussian blob appeared more to the left or more to the right of the bottom one. Each subject performed 150 trials with leftward moving dots in the upper subwindow and 150 trials with rightward moving dots in the upper subwindow (i.e., 300 trials). Thus, there were 10 conditions: 2 motion directions (i.e., upper field leftward and bottom field rightward, and vice versa) × 5 offsets; each condition was repeated 30 times. Fast and slow coherently moving dots were displayed in separate blocks: (ii) a control condition, identical to the experimental condition but without moving dots. In this condition, subjects performed 150 trials (i.e., 30 repetitions per each offset); (iii) a further control condition in which dots moved randomly (0% coherence). In this condition, subjects performed 150 trials (i.e., 30 repetitions per each offset). The latter session served as control for the influence of motion background on the perceived spatial position of the two test Gaussian blobs. Fast and slow coherently moving dots were displayed in separate blocks. Method of Constant Stimuli (MCS) was used for all conditions. A logistic function [24] was fitted to the data in order to estimate the 50% corresponding to the physical misalignment between the Gaussian blobs required for apparent alignment (the point of subjective equality, PSE [25, 26]) (see [26, 27] for a similar procedure).

## 3. Results

**Figure 2:**
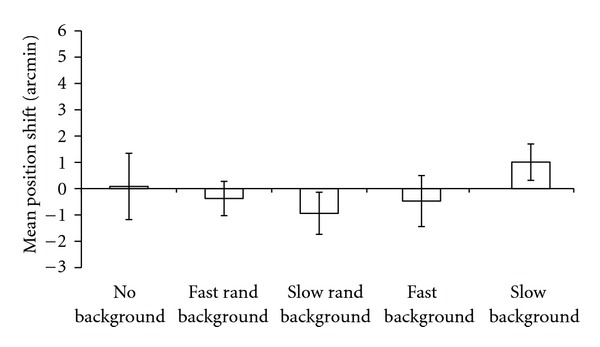
Mean PSEs (*N* = 11). A one-way repeated measures ANOVA did not point out any significant effect of the background type on the PSEs. Error bars ± SEM.

PSEs and Slopes were calculated individually for each subject. All curve fits passed a *χ* ^2^ goodness-of-fit test. Overall, the results showed that background motion had no effect on the perceived position of stationary Gaussian blobs. A one-way repeated measures ANOVA did not point out a significant effect of the background type (*F*(4,40) = 0.75, *P* = 0.41, and *η* ^2^ = 0.07). In addition, simple contrasts did not point out any significant difference between the different Types of background. Moreover, a series of Bonferroni corrected-one-sample t-tests did not revealed any significant difference between any of the background types with respect to zero (Figure 2).

Thus, neither fast nor slow moving dots influenced the perceived position of stationary flashed objects.

**Figure 3:**
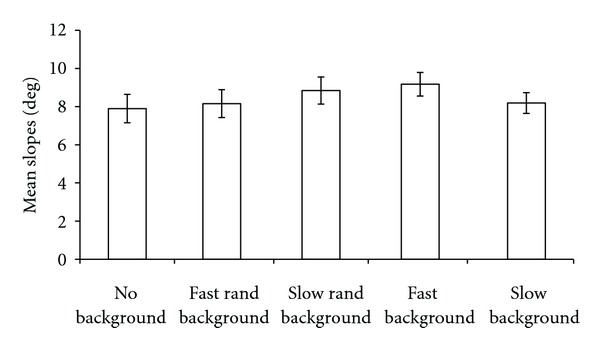
Mean slopes (*N* = 11). The slopes were calculated as the reciprocal of the standard deviation of each psychometric function. A one-way repeated measures ANOVA did not point out any significant effect of the background type on the slopes. Error bars ± SEM.

Figure 3 shows the mean Slopes of the best-psychometric functions. The slopes were calculated as the reciprocal of the standard deviation of the psychometric function. In this case too, a one-way repeated measures ANOVA did not point out a significant effect of the background type on the slopes (*F*(4,40) = 1.68, *P* = 0.17, and *η* ^2^ = 0.14). Simple contrasts did not point out any significant difference between the different types of background. The absence of a significant difference between the slopes indicates that there were no differences in subjects' ability to discriminate small differences in position across the different background conditions [27].

## 4. Discussion

In the present study, we investigated whether moving dots at a speed that produced motion streaks (i.e., motion smear) affected the perceived position of two flashed stationary Gaussian blobs. Based on Geisler [1], Geisler et al. [2] and Edwards and Crane [6], we used coherently fast (17.7 deg/s) moving dots that produced motion streaks and coherently slow (1.77 deg/s) moving dots that did not produce motion streaks. Motion streaks influence the perceived direction and speed of moving stimuli [3, 7], but our results show that they do not influence the perceived position of stationary objects.

There is psychophysical evidence that motion streaks can affect, under certain stimulus conditions, the shape of illusory contours. Li et al. [9], for example, have shown that when a field of fast (6 deg/s) globally contracting moving dots (diameter: 5.4 arcmin) was superimposed to a convex Kanizsa triangle, the edges of the Kanizsa triangle were perceived as regular. Thus, the contracting dots induced a distortion of the illusory edges of the Kanizsa triangle along the motion direction. However, Li et al. [28] showed that using a motion-defined contour, the presence of fast moving dots (speed > 2.5 deg/s) in the background did not affect the perceived shape of the motion-defined contour. In particular, the authors investigated how complex motion patterns affected the perception of shape. They used two superimposed fields of globally moving dots; one field moved along an ellipsoidal trajectory (i.e., target), while the other field was split into quadrants with dots in alternating sectors moving either in radial expanding or radial contracting directions (i.e., background motion). The results showed that the appearance of the ellipsoidal target was not distorted in the presence of background motion. However, the distortion was observed only at slow background speed, and when dots had high contrast and were of the same luminance polarity. It should be noted that in the case of Li et al. [28] the motion defined shape of the object was always clearly discernable, and the speed at which moving dots affected the shape of the ellipsoid was below the critical speed for motion streaks. Indeed, dots diameter was ~6 arcmin and, based on Geisler [1], for ~6 arcmin dots, streaks start to emerge at a speed of ~2.5 deg/s. Thus, it is likely that motion streaks cannot affect the shape of discernable objects, but they do when the contours are not defined providing a spatial signal that might reduce spatial uncertainty.

We found that motion streaks do not bias the perceived spatial position of stationary objects with fuzzy contours (i.e., Gaussian blobs), suggesting that the spatial and motion signals carried by motion streaks do not interact with the spatial representation of the Gaussian blobs. Thus, it is possible that motion streaks present in the background do not unbalance the perceived contrast between the two edges of the Gaussian blobs, preventing a shift of the centroid of the Gaussian blobs in the direction of motion [20, 21].

This could reflect the presence of a deblurring/sharpening mechanism occurring at high velocities that limited the motion smear and thus the extraction of motion streaks [4]. Bex et al. [29], for example, proposed a task in which observers were required to match a drifting grating with a blurred static grating, in order to measure the effect of velocity on the perceived blur of a drifting grating; they found that the gratings appeared less blurred when drifting than when stationary; thus, the drifting grating was deblurred. Moreover, when increasing the velocity of the drifting grating, its perceived blur decreased, meaning that deblurring increased with speed (see also [30] for similar results).

When using fast moving dots, the fact that Gaussian blobs were flashed after 150 ms from the beginning of the dots' motion could have limited the extraction of motion streaks. Burr and Morgan [31], indeed, have shown that briefly presented moving stimuli (e.g., 40 ms) appeared more blurred than longer presented stimuli (e.g., 150 ms). The reason is that moving dots may activate mechanisms tuned for motion and mechanisms that encode static signals [32]. The motion mechanisms encode motion and should not signal blur [29, 33], whereas mechanisms for static vision should integrate contrast energy over time and respond to the (integrated) streak caused by motion. Moreover, a brief motion signal has a reduced motion energy in the veridical direction and additional motion energy in the opposite direction, due to the increased spread of temporal frequencies [34, 35]. Thus, the contribution of the motion response relative to the nonmotion response rapidly increases with time. If signals from motion detectors are “*blur-free*”, then apparent blur should decrease with exposure duration. Thus, in our casem the high velocity and the relatively long exposure to directional motion may have limited (or prevented) the extraction of motion streaks in the condition with fast moving dots and thus the resulting weak motion streaks (i.e., motion smear) were not sufficient to shift the centroid of the Gaussian blobs, thus inducing MIPS.

However, Khuu et al. [10] found that the shape of a Gaussian blob flashed in the periphery of the visual field and during an apparent motion sequence was perceived wider than the reference Gaussian blob and slightly shifted in the direction of the apparent motion sequence. It is not clear yet the mechanism/s involved in such distortion (and positional shift) during apparent motion, but they are likely to reflect high-level interactions between motion and form [10]. To some extent, it is possible that motion smear arising from apparent motion is not deblurred or the deblurring process takes long time to be effective.

In summary, our results showed that background motion did not influence the perceived spatial position of stationary flashed objects. Motion streaks, arising from fast moving feature, are able to influence only the shape of illusory contours [9], where the spatial uncertainty is very high. However, motion streaks do not influence the perceived spatial position of discernable objects (e.g., Gaussian blobs or motion defined contours [28]) by inducing local distortion of the shape. In addition, our data support the notion that motion blur and motion deblurring/sharpening mechanisms constantly interact.
